# ‘*It Is Like a Cross‐Stitch … All Joined Together But Not Making a Very Nice Pattern’*: A Qualitative Study of Patient Perspectives on Physical Health Inequalities in Severe Mental Illness

**DOI:** 10.1111/hex.70367

**Published:** 2025-08-06

**Authors:** Tassia Kate Oswald, Stan Papoulias, Julie Williams, Jayati Das‐Munshi

**Affiliations:** ^1^ Department of Psychological Medicine, Institute of Psychiatry, Psychology & Neuroscience King's College London London UK; ^2^ Population Health Improvement UK (PHIUK), United Kingdom UK; ^3^ Centre for Mental Health and Community Wellbeing, Melbourne School of Population and Global Health University of Melbourne Melbourne Australia; ^4^ Service User Research Enterprise, Institute of Psychiatry, Psychology and Neuroscience King's College London London UK; ^5^ Centre for Mental Health Policy and Evaluation, Institute of Psychiatry, Psychology and Neuroscience King's College London London UK; ^6^ ESRC KCL Centre for Society and Mental Health London UK; ^7^ South London & Maudsley NHS Foundation Trust London UK

**Keywords:** barriers, comorbidity, physical healthcare, qualitative, service use, severe mental illness

## Abstract

**Background:**

People with severe mental illness (SMI) have worse physical health than the general population and face a range of challenges with their healthcare. This study aimed to explore the interplay of issues across system, service, social and individual levels that impact healthcare experiences and outcomes from the perspective of people with comorbid SMI and physical health needs.

**Methods:**

A qualitative study was conducted. Fifteen individuals participated through focus groups and interviews (mean age = 55 years; range = 39–74 years). Participants were secondary mental health service users living with comorbid SMI and physical health condition(s) in South East London, the United Kingdom. Data were analysed through a process of inductive thematic analysis with reflexive elements.

**Results:**

Seven themes were generated, demonstrating the interplay of system, service, social and individual‐level issues. The first two focused on prevailing issues described by participants, including ‘systemic barriers to effective and equitable social and health care’ and ‘interpersonal stigma exacerbating inequalities’. The third and fourth themes described the consequences of these issues: ‘services turning to medication in response to multiple constraints’ and ‘inequities limiting individual actions’. Themes 5 and 6 centred on creating equitable conditions for people with SMI and physical healthcare needs, including ‘the role of informal and formal social supports’ and ‘patient perspectives on what works in health services’. The seventh cross‐cutting theme described the ‘intertwined nature of mental and physical health’.

**Implications:**

This study highlights systemic barriers and interpersonal stigma as sources of inequality for people with comorbid SMI and physical health needs. The findings highlight the need for investment in social and healthcare roles, services and systems which can be responsive to compounding risks experienced by these individuals. Both physical and mental health needs, and their interactions, should be considered, along with the cumulative impact of poor socio‐economic conditions and stressors.

**Patient or Public Contribution:**

A service user steering group, comprising five people with lived experience of SMI, was convened to support the study. The group met to inform the development of research questions, topic guides and participant materials, support the interpretation of findings, and provide input on the paper.

## 1 Introduction

Severe mental illness (SMI) is an umbrella term incorporating several persistent psychiatric disorders, including schizophrenia, bipolar disorder and psychotic disorders, which can result in serious functional impairment and psychological distress [1, 2]. It is well established that people with an SMI diagnosis also have poorer physical health than the general population, including increased rates of cardiovascular disease, respiratory disease and diabetes, leading to a reduced life expectancy [3, 4, 5]. The causes for this are complex, spanning system, service, social and individual levels.

At a system level, the physical health of individuals with an SMI diagnosis is impacted by wider socio‐economic determinants [6, 7]. Examples include higher levels of unemployment [8] and impacts of welfare reform and conditionalities [9, 10]. While excess mortality in those with SMI occurs across all socio‐economic quintiles, it is most marked in the most deprived quintiles when suicide is excluded as a cause of death [11], with chronic stress potentially mediating associations [12].

At the service level, inequalities in healthcare provision may also play a part, including less assertive treatment of physical health conditions in people with SMI and fragmented physical healthcare across primary care and secondary mental health services [13, 14]. While physical health should be monitored in primary care in people with established psychosis, primary care staff may not feel confident working with people with SMI [15], and, reciprocally, secondary mental health staff do not always feel confident in identifying and managing physical health problems. Perceptions of professional ‘boundaries’ may ultimately impact on how far clinicians are willing to intervene in conditions perceived to be outside of their remit [16, 17]; indeed, mental health service users frequently report the challenges of seeking physical healthcare across healthcare services arranged in vertical siloes [7].

On a social level, discrimination and stigma associated with SMI can also affect physical healthcare and outcomes. For example, discrimination and stigma may involve dismissing patient reports of physical health morbidity, or wrongly attributing them to a mental health condition when they present in someone with a known mental illness diagnosis, known as ‘diagnostic overshadowing’, which further compounds the situation [18].

At the individual level, behavioural risk factors for cardiovascular disease are increased in individuals with an SMI diagnosis, such as smoking/tobacco use, substance use and higher levels of sedentary behaviours [19], which may be partly due to the negative symptoms of schizophrenia. This is further compounded by psychotropic medications, which have been linked with increased risk of obesity, metabolic issues, diabetes and cardiovascular disease [20].

While there is a growing evidence base which describes challenges in healthcare and related physical health outcomes for individuals with SMI diagnosis [21], the literature is fragmented and often overlooks the interplay of these system, service, social and individual‐level issues. There has been a longstanding emphasis on self‐management and behaviour modification approaches for this population; however, these approaches overlook upstream factors that shape healthcare experiences and outcomes.

The aim of this study is to explore the interplay of issues across multiple levels that impact healthcare experiences and outcomes, from the perspective of individuals with comorbid SMI and physical health needs, with a further focus on the consequences and possible solutions to these issues.

## 2 Methods

To address the aim of our study, a qualitative study was conducted.

### 2.1 Participants and Setting

Study participants were people living with SMI who had previously or were currently under the care of a large Mental Health National Health Service (NHS) Trust, the South London and Maudsley (SLaM), which serves an ethnically diverse population of 1.3 million people in South East London, the United Kingdom. To be eligible for participation in the study, individuals needed to have been over the age of 18 years and had a diagnosis of an SMI, defined as the presence of a clinical diagnosis of schizophrenia spectrum, bipolar disorders or non‐organic psychotic disorders. Individuals also need to currently be managing a physical health condition to be eligible (e.g., diabetes and respiratory conditions). Any comorbid long‐term physical health condition was eligible for inclusion, and there were no criteria relating to length of time since onset.

Individuals were not eligible for participation if they were currently an in‐patient on a mental health ward, under the care of acute crisis teams (e.g., the Home Treatment Team) or detained under the Mental Health Act, if they were unable to give informed consent to take part in the study, or if significant concerns had been raised relating to risk.

### 2.2 Recruitment Approaches

Two approaches were employed to purposively sample participants to take part in focus groups or interviews. In the first approach, we used *Consent for Contact (C4C)* [22], which is a registry of people using mental health services under the SLaM Trust who consented to being contacted for research. Using the C4C register, we searched for participants with relevant SMI diagnoses according to ICD‐10. This included people with schizophrenia‐spectrum disorder diagnosis (F2*) or bipolar affective disorders (F30, F31). Using this approach, we also used search terms related to diabetes and respiratory health conditions (e.g., COPD) to identify participants with comorbid physical health conditions. For all service users currently on the C4C register, care coordinators were contacted first to ensure there were no concerns about approaching the individual. In addition, clinical records were screened to ensure that service users were not actively in crisis and/or admitted to inpatient units, before being sent invitations to participate. In the second approach, a member of the research team (J.W.) approached individual community teams in the mental health Trust via email and presented details of the study, inviting them to display study posters and inform service users under their care if they wished to take part. The posters specified that the study was about physical health needs and care for people with a severe mental health diagnosis.

Potential participants identified were contacted by either a member of the research team or a Clinical Studies Officer who supported study recruitment and was employed by the Mental Health Research Network. Potential participants were given details of the study and the opportunity to ask questions. If they were interested in participating, individuals were invited to take part in a focus group or an individual interview. All participants were provided with information sheets and consent forms. Informed written consent was obtained before participating in the study, and participants were informed of their right to withdraw at any time. All participants completed a short demographic questionnaire.

### 2.3 Patient and Public Involvement

A service user steering group, comprising five people with lived experience of severe mental health challenges, was convened to support the study. The group met to inform the development of research questions, topic guides and participant materials (information sheets and posters) and to support interpretation of findings from the data. The advice of the steering group led to modifications in participant materials and in study design and conduct. For example, the group advised on methods to recruit more diverse participants, which were subsequently followed, improving the diversity of study participants. Following the group's feedback, individual interviews were offered alongside the possibility of participating in focus groups to ensure that study participants had a choice in how to participate, especially if they felt less comfortable speaking in a group setting. The steering group also advised on the wording of the topic guides, particularly around sensitive topics such as asking about experiences of discrimination. As a result, subsequent wording used in the topic guide specifically enquired about experiences related to diagnostic overshadowing or experiences of discrimination due to other protected characteristics. Furthermore, preliminary findings and emergent themes were shared and discussed with the group to increase confirmability [23]. Finally, the team presented a complete draft of this manuscript and a plain English summary to the steering group and incorporated their comments into the final version.

### 2.4 Focus Groups and Interviews

Focus groups and interviews were conducted with participants. Focus groups were convened in community settings and in university seminar rooms with light refreshments provided. They were co‐facilitated by two clinical researchers (J.D. and J.W.) with experience working with people with SMI. During each group, either J.D. or J.W. would lead the discussion, with the other person observing and taking notes. Focus groups lasted for approximately 90 min.

Interviews were conducted by either J.D., J.W. or S.P. They lasted between 60 and 90 min and were conducted in community or university rooms. Later interviews were conducted online or by telephone due to lockdown restrictions during the Covid‐19 pandemic.

Focus groups and interviews followed the same topic guide. Participants were encouraged to expand on each of the topic areas within the guide, which sought to explore issues experienced by participants across system, service, social and individual levels. Topics included: barriers to accessing healthcare, communication and coordination among providers, desired changes in healthcare services, experiences of discrimination or unfair treatment, experiences with healthcare providers, facilitators for managing physical health, influence of personal characteristics on care received, perceived understanding by professionals, personal strategies for physical well‐being, and sources of healthcare support.

### 2.5 Socio‐Demographic Data

Participants were asked to complete a brief socio‐demographic questionnaire, which asked for details about their age, gender, ethnicity, sexual orientation, mental health diagnoses and physical health conditions.

### 2.6 Data Analysis

Focus groups and interviews were recorded on a digital voice recorder and transcribed by a transcription agency, with transcripts checked against tapes by J.D. and J.W. to ensure accuracy. NVivo software was used for coding, and data were analysed using thematic analysis [24]. T.K.O. read through all transcripts to become familiar with the content. Data was then analysed by T.K.O. through a process of inductive thematic analysis [24] in which codes were derived from the data across three rounds of coding and shared with the other authors for further iterations and to ensure completeness and representativeness of the codes. Codes were assigned to emergent themes, which were discussed and further refined following feedback from other authors. Data collection and analysis proceeded until saturation was achieved.

### 2.7 Ethics Approvals

Ethics approval was obtained from the London‐Camberwell St Giles Research Ethics Committee (REC reference [18]/LO/0056) to conduct this study. Service user participants were reimbursed £20 for focus groups and £15 for interviews, plus reasonable expenses for travel, consistent with NIHR‐approved rates at the time of the study.

## 3 Results

Fifteen individuals participated in the study through two focus groups (*n* = 3 and 4 participants, respectively) and eight individual interviews (*n* = 3 in‐person and 5 online). Responses to the socio‐demographic questionnaire were limited, but the available data indicate that participants were male (*n* = 7) and female (*n* = 8), a third of the sample reported they were White British (*n* = 5; unknown for *n* = 3), mostly heterosexual (*n* = 9; unknown for *n* = 4), and aged 55 years on average (range = 39–74; unknown for *n* = 3).

All participants described comorbid physical health and mental health diagnoses and were under the care of the SLaM secondary mental healthcare provider at the time of the study. Bipolar disorder was the most commonly reported SMI diagnosis (*n* = 7), and participants additionally reported diagnoses of agoraphobia, anxiety, attention deficit disorder, emotionally unstable personality disorder or borderline personality disorder, major depressive disorder, post‐traumatic stress disorder, schizoaffective disorder, and schizophrenia. Diabetes mellitus was the most commonly reported physical health condition (*n* = 5), and participants additionally reported managing alopecia, anaemia, asthma, brain injury, chronic obstructive pulmonary disease, chronic sinusitis, high blood pressure, high cholesterol, migraines, polycystic kidneys, sciatica or back pain, stomach ulcers, thyroid issues, uterine fibroids, and other unspecified injuries.

**Figure 1:**
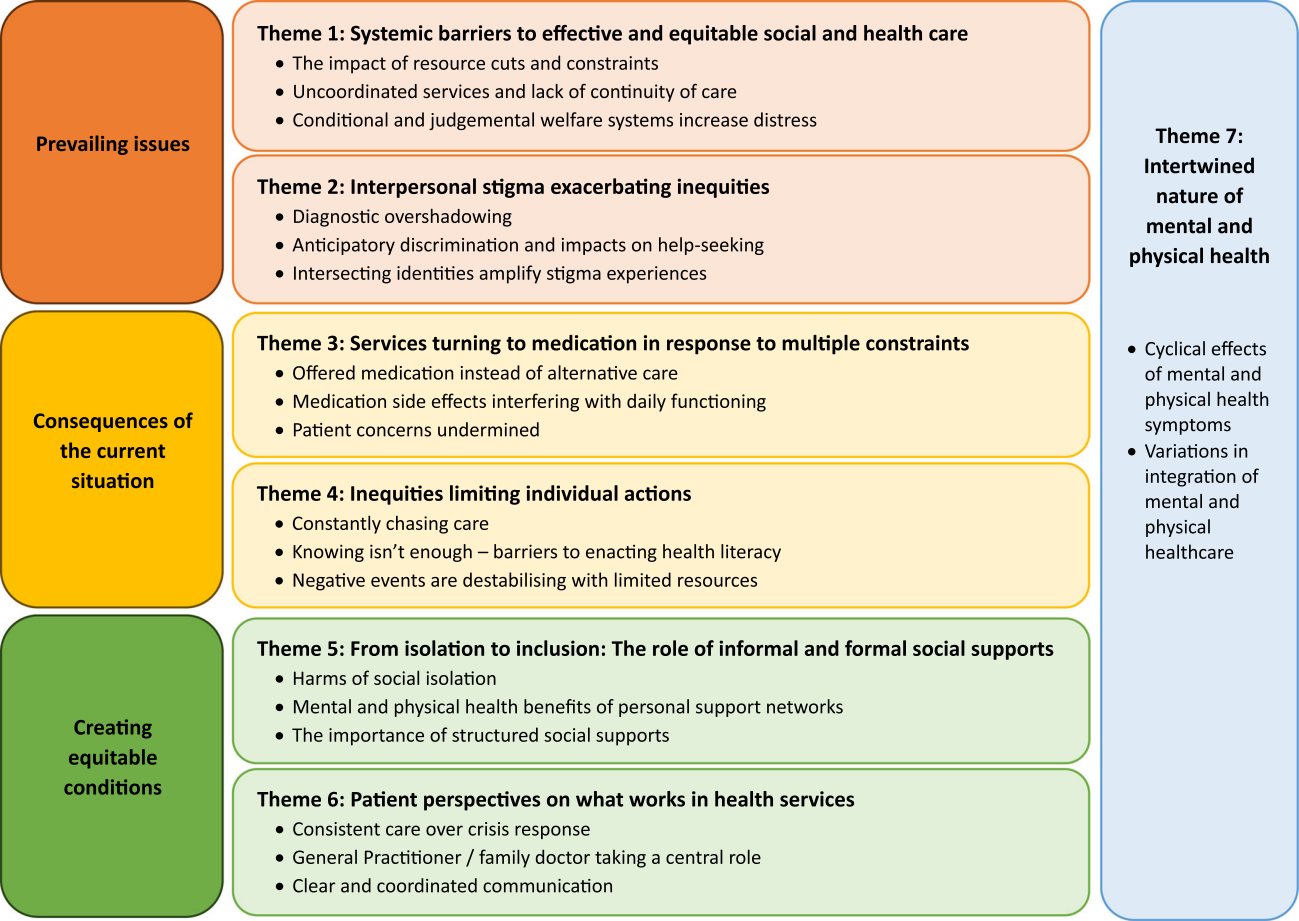
Map of themes and sub‐themes generated through thematic analysis.

Figure 1 presents a map of the thematic analysis, which includes seven generated themes demonstrating the interplay of system, service, social and individual‐level issues. The first two themes focus on *prevailing issues* described by the participants; the third and fourth themes focus on the *consequences* of these issues; and themes 5 and 6 centre on *creating equitable conditions* for people with SMI and physical health needs. The seventh theme describes the intertwined nature of mental and physical health, which is a cross‐cutting theme. Accompanying qualifiers are used in the results to signify the data collection mode and number for a quote (FG = focus group; I = interview).

### 3.1 Theme 1: Systemic Barriers to Effective and Equitable Social and Healthcare

Participants described a range of systemic barriers to effective and equitable social care and healthcare. Foremost, participants commented on experiencing uncoordinated health services and a lack of continuity, which impacted the care they received. Several participants described rotating through numerous health professionals and not making any progress with their health due to a constant need to update new doctors on their situation, which was considered a waste of time and resources.

‘I think I saw about nine psychiatrists: I might see them once and then they would disappear, the same with the keyworker. Even my GP said, “Which number are you on now?” and it was totally pointless’ (FG1).

‘The worst thing is going to a doctor, them not knowing you, and having to be saying, “I've got bipolar, I've got EUPD^1^, I've got this, I've got that,” then it is, “There's not enough time for this appointment. Sorry, you'll have to make another”… I just wish that sometimes you didn't have to go and explain every single thing.’ (I2).

Most participants identified that the lack of coordination and continuity experienced in their healthcare at the service level was largely due to system‐level issues like resource cuts and constraints in the NHS. Austerity and political decisions were seen to affect the budget for mental health, leading to overstretched and underpaid staff who were unable to provide proper care.

‘But there is always talk about doctors haven't got enough time, they haven't got enough money. GPs are leaving the country’ (FG2).

‘I think it is a political thing for me. I think you can't turn off mental illness when you have got mental illness. So, I was heavily against the Conservatives cutting back on the budget for mental health’ (FG2).

Beyond healthcare, participants spoke about conditional and judgemental welfare systems, which, on a system and social level, discriminated against people with mental illness and were distressing to navigate. The act of seeking financial support was described as more stressful than attempting to remain in employment when unwell, particularly when needing to prove one's fluctuating health status and meet conditionalities.

‘Don't make it so hard. Don't make the forms so confusing…. It is a fight. And it was much easier when I was working and just ignoring my mental health and just running myself into the ground…. They need to understand, the government, that a fluctuating condition with mental health along with a fluctuating physical condition is a very bad combination, and you can't keep track of it’ (I2).

### 3.2 Theme 2: Interpersonal Stigma Exacerbating Inequities

More proximally, a range of interpersonal stigma experiences were described by participants on a social level, which further exacerbated inequities in their healthcare on a service level. Participants recounted experiences of ‘diagnostic overshadowing’, in which their physical health symptoms were dismissed as being a part of their mental illness. As a result of these experiences, participants often anticipated discrimination from health professionals, feeling that they would not be taken seriously, which affected timely help seeking.

‘Too many of my symptoms get brushed off as part of my [mental] illness, when they are actual physical elements to what is happening’ (FG2).

‘If you have got a long record of mental health admissions and problems … they might not listen as intently as they would to a member of the public who has had no issues’ (I5).

‘In a way, that sometimes stops me seeing the GP about things, because I think I will be blamed’ (I4).

In addition to stigma associated with mental health diagnoses, participants described amplified stigmatising experiences related to various intersecting identities, which may have compounded disadvantage. For example, some individuals discussed the impacts of being an older person, or from a racially minoritised group, on their interactions with people and services.

‘All of a sudden they [charity organisation] wanted younger people, so they put me on a shelf’ (FG1).

‘I find, being a black person in England, there are issues…. Having a white [care] coordinator doesn't really help me because he doesn't have the sympathy to deal with racial problems’ (FG2).

### 3.3 Theme 3: Services Turning to Medication in Response to Multiple Constraints

In response to the prevailing systems‐level constraints described, all participants indicated that medication was frequently turned to as a solution on a service level. For example, participants were often offered medications instead of preferred forms of alternative care, such as psychotherapy, due to unavailability of appointments.

‘You don't get offered anything else that might help. It is just, “We'll try this medication.”’ (FG1).

‘[The health service] were like, “No, we can't fit you in for another appointment. We'll send you a prescription through the post for another med,” and, to me, that just wasn't good enough, because the other meds weren't working’ (I2).

The use of medication as a response to multiple constraints ultimately had consequences for patients on an individual level. Lack of continuity of care and inadequate communication about medications resulted in constant medication changes and side effects, which interfered with their daily functioning. Some participants even commented that they were worse off on medications.

‘Some of my medication has affected my physical health…. I was on quite high doses of lithium for a treatment for depression…. I got hypothyroidism and chronic kidney disease’ (I4).

‘The doctor had to intervene with the psychiatrist over medication, which, to me, was life‐threatening symptoms’ (FG2).

‘Every time I get a different doctor, they decide to change my meds’ (I6).

Most participants expressed that their concerns about medications were repeatedly undermined by health professionals. In some cases, this resulted in years of suffering through debilitating side effects and frustration for not being listened to.

‘I am saying this is the problem—I started taking these tablets and I was worse than before. And he took no blind bit of notice and ignored what was being said by me, the patient’ (I3).

‘I am sorry that they didn't take my concerns over clozapine a lot more seriously, because I think I had 20 wasted years of my life’ (I5).

‘I told the doctor, “You don't listen to me.” I told the psychiatrist, “You don't listen to me. I told you I was better on this drug,” and then she said, “OK, I agree with you.” So, she actually disturbed my life for 2 months, and I couldn't work, I couldn't do anything’ (FG1).

### 3.4 Theme 4: Inequities Limiting Individual Actions

Participants discussed a range of inequities they experienced across multiple levels of influence, which affected how they managed their own health. For example, in under‐resourced settings, participants often needed to advocate for themselves to receive appropriate care. Constant follow‐up about medications, appointments, referrals and results was described as added stress, and something which not everyone had the ability or capacity to do. While some participants explained that they were receiving all the care they needed, they expressed difficulty in reaching that point.

‘Constant phoning, and then it is just irritating the receptionists. What if I was very elderly and not able to? It is not working. They are supposed to do it electronically, but, in the end, the GP said, “Just put them in by hand,” but, again, it is just more work to do’ (FG1).

‘It is like we have to help ourselves. They are supposed to be helping us, but we are helping ourselves’ (FG1).

‘I am getting it all, but it has taken me a lot of shouting, a lot of mental health anguish and several mini breakdowns to actually get that’ (I2).

Notably, all participants demonstrated strong health literacy and were able to describe what behaviours were needed to maintain a baseline of wellness at the individual level, yet, despite this, they concurrently described a range of social and system‐level barriers to enabling this. In particular, social and economic inequities were limiting when it came to being able to afford healthy foods or attend health appointments. This highlights that simply *knowing* was not enough; there is a need to address upstream factors instead of placing all the onus on individuals through behaviour modification approaches.

‘Some people haven't got the money if they fall sick. They haven't got the support to say they can buy nutritious food as aid in their recovery’ (I3).

‘I didn't go there [to health appointment] for about a month…. I hadn't got my PIP^2^ money through, so I couldn't afford the taxi fare and stuff there’ (I2).

Participants also articulated compounding difficulties in taking care of themselves while managing mental health lows related to their SMI. Mental health and resource inequities experienced by individuals with SMI and physical healthcare needs can also mean unexpected negative events may be destabilising and difficult to cope with. For example, external influences, like maintenance issues in the home, can act as additional setbacks, which may destabilise participants, thus making them more likely to resort to unhealthy coping strategies despite knowing that they are unhealthy.

‘When I am going through my depression, I can't exercise. I can't get out of bed, I can't do anything. I just want to stay in bed’ (FG1).

‘It is very much entwined with my mental health and, if there is stress or difficult emotions around, I find some emotions quite difficult and, when that is happening, I tend to eat biscuits or ice cream or cake or whatever, even though I know somehow or other that I feel better if I eat healthy’ (I4).

‘It is called external influencers. When I am trying to get myself a little bit more sorted, all of these things come crashing down and then it makes me feel like I am making absolutely no progress whatsoever’ (I2).

‘You can't cope with stress in the same way that other people can’ (FG1).

### 3.5 Theme 5: From Isolation to Inclusion: The Role of Informal and Formal Social Supports

At the social level, the role of informal and formal social supports was described by most participants as key to creating equitable conditions for people with SMI and physical health needs. Participants described the harms of social isolation, as being disconnected could lead to a lack of perspective on one's thoughts and feelings. On the contrary, informal, personal support networks were seen to have benefits for both mental and physical health. Connecting with friends allowed individuals to receive psychological feedback and was also an opportunity to be physically active through getting out of the house, walking and engaging in activities. In the absence of personal social supports, participants spoke about the importance of structured social supports and activities at the service level, for example, through charities.

‘I like not to get isolated. So, I try to help my friends as much as possible, which gives feedback…. If I don't have the sociable aspect, I get ill and I start to get withdrawn and my thinking goes a bit’ (FG2).

‘I am around people and I get to do computer work and some database work and I get to do reception, and that keeps me always in contact with people and not so isolated on my own in my room’ (FG2).

‘They [charity] have walking groups which they are trying to help me with. So, the support is very good’ (I5).

### 3.6 Theme 6: Patient Perspectives on What Works in Health Services

Despite prevailing issues described by participants, several perspectives and examples of what works to create equitable conditions in health services were discussed. Importantly, participants emphasised their preference for, and the benefits of, consistent care over and above support confined to crisis situations. One participant commented that, ‘*if somebody is at death's door, they take priority’ (FG2)*, while other participants elaborated on the benefits of receiving consistent care:

‘[The health service] have helped me, not only with the psychiatrist part of things, having regular psychiatry input instead of just once every 7 years because you are in a crisis…. I feel there is continuity now’ (I2).

‘The doctor, now I am getting to know her and she is getting to know me, because we have demanded some continuity, which is effectively more for my mental health than everything’ (I1).

Participants described successful healthcare management when their GP took a central role in their care, and many participants spoke about positive experiences with their GP. They discussed the benefits of the GP making helpful referrals, providing health knowledge/literacy, signposting for check‐ups, and generally being their ‘*first port of call’ (I5)*, particularly for physical health concerns.

‘Every year they make sure they call me to come over there and they do a physical check up on me: blood pressure, all of the stuff’ (I6).

‘He [GP] was good enough to take a real interest and he spent a while on the phone and then made an appointment for me…. He [GP] went out of his way to make sure I was seen and I got seen by somebody and it was great’ (I5).

Clear and coordinated communication between health professionals about mental and physical health needs further improved participants' healthcare experience, often with the GP acting as the middle person in these situations.

‘When I go to have my diabetes eye check, it comes from the GP, so they send all the results to the GP. So, they are communicating with lots of people always’ (FG2).

‘All the information goes to the GP. So, they communicate with the GP and the GP communicates with me’ (FG2).

### 3.7 Theme 7: Intertwined Nature of Mental and Physical Health

The intertwined nature of mental and physical health was repeatedly discussed by participants, ‘*because they go hand in hand’ (I6)*. In particular, the cyclical effects of mental and physical health symptoms on an individual level featured in conversations, as participants spoke about how physical health symptoms would trigger mental health symptoms and vice versa.

‘When things go wrong with your physical health, you start to worry, and the worry can trigger off very, very serious mental health.’

Participants shared varied experiences in the integration of mental and physical healthcare they received at the service level. Some participants reported positive experiences in which physical health professionals took their mental health seriously, often acting ‘*like counsellors as well’ (I8)*, or other instances when mental health professionals took individuals' physical health into account.

‘I think they took it into account that it played a part in my mental health as well … in the endocrine clinic, the thyroid nurses are always commenting on, “I see this affects your mental health as well,” and she took that very seriously. I think there was a great connection between them…. They always commented on, “If your thyroid is out of control, then your mental health gets out of control, so it is very important to keep your thyroid in control to keep your mental health in control.” So, it was great’ (I5).

Other participants described barriers at the service level, with their mental health viewed as being out of remit or the skillset of some doctors:

‘When I am not feeling that great and I go, they [physical health doctors] don't really pick up on it, but it is not their forte, is it, to pick up on mood swings’ (I1).

‘My physical health my GP is not too bad with, but there is this cut‐off; it is like you are not dealt with holistically’ (FG1).

## 4 Discussion

There is a growing evidence base which describes challenges faced by individuals with comorbid SMI and physical health needs, but the literature is siloed and often overlooks the interplay of issues across multiple levels of influence. This qualitative study sought to address this gap by exploring the interplay of system, service, social and individual issues, from the perspective of people with comorbid SMI and physical health needs. Participants identified current issues in healthcare experiences and outcomes, discussed the consequences of these issues, and proposed the creation of equitable conditions to address these issues moving forward.

Our findings highlight that this group are affected by a range of systemic barriers to effective and equitable social care and healthcare, as well as experiences of more proximal interpersonal stigma, which further exacerbate inequalities in care and outcomes. In response to these systemic issues, participants indicated that medication was frequently turned to as a solution. Inequities in system‐ and social‐level factors further limited the individual actions participants could engage in to support their own health. Both informal and formal social supports were viewed as central to maintaining overall health, along with consistent healthcare with a regular GP/family doctor taking a central role. Across the board, participants reiterated the intertwined nature of mental and physical health, especially the cyclical effects of mental and physical health symptoms and reported varied experiences in the integration of mental and physical healthcare they received.

Our findings resonate with recent qualitative research focusing on self‐management in people with SMI and chronic conditions in the United Kingdom. For example, Carswell et al. [25] discuss the circularity of symptoms related to SMI and long‐term conditions and highlight patients' tendency to normalise defaulting to maladaptive coping strategies during acute episodes of distress, at the detriment of their physical health. In another study, patients similarly expressed that their capacity to self‐manage their diabetes was governed by their symptoms [26]. It has previously been reported that living with an SMI may be associated with a higher prevalence of maladaptive health behaviours, reduced self‐care behaviours and decreased help‐seeking—an amalgamation which increases the risk of developing and worsening physical health problems [27]. However, individuals in our study emphasised that their health behaviours were not simply a choice; rather, they were affected by not only the symptoms of SMI, but also by system and social‐level factors.

Our study extends beyond themes related to self‐management and health behaviours by highlighting the broader social, structural and economic contexts in which participants reflected that they were constrained by. A notable strength of the present study was our diverse sample (one‐third White British) and the topic guide, which allowed us to gain insight into how issues like stigma, discrimination and inequities operate at systemic, service, social and individual levels. We go beyond considering multimorbidity through purely biomedical and behavioural lenses, ensuring we do not sideline the important contribution of wider socio‐economic, systemic and stigmatising factors to healthcare and outcomes [28]. Our findings underline a need to move beyond single disease frameworks, to capture and understand the broader determinants, which cumulatively impact mental and physical health and highlight the need to address upstream factors instead of placing the onus on individuals through behaviour modification approaches.

Participants in our study described the negative impacts of systemic barriers to effective and equitable healthcare. Lack of continuity in care was expressed as a central problem. This is particularly concerning given a recent review cited continuity of care as a key ingredient to preventing premature deaths, lowering emergency visits and contributing to better quality of life in people with SMI [29]. In line with this, most participants in our study expressed positive healthcare experiences when their GP/family doctor took a regular and central role in their care. However, our participants also acknowledged that GPs are stretched too thin, and with increasing expectations on GPs to manage mounting components of their patients' health and well‐being, it is important that adequate resourcing and expectations are set out to support primary care provision [30].

Participants further expressed the impacts of stigma, and particularly the impact of diagnostic overshadowing experienced in services. Reflective of this, a UK study, which aimed to determine the prevalence of 24 chronic physical conditions in people with SMI, reported that some conditions were under‐recorded for schizophrenia patients [4], suggesting physical health conditions are not being adequately captured for intervention in this population. A further qualitative study highlighted the direct adverse impacts of diagnostic overshadowing on people with SMI presenting to emergency departments, leading to delays or failure in receiving potentially life‐saving treatments [18]. In addition, participants in our study described the harms of social isolation, alongside the importance of social support, on their mental and physical health. Other literature reinforces the harms of social isolation, with a recent study reporting associations between a measure for ‘social disconnectedness’ (comprising low social support, isolation and loneliness) and mortality across mental health conditions, including SMI [31].

Together, the qualitative findings from our study, along with quantitative evidence across the literature, reinforce the influential role and interplay of system, service, social and individual‐level factors, which create inequalities and exacerbate poor health outcomes in individuals with comorbid physical health needs and SMI. There have been calls for care which is more integrated and involves more holistic responses to multimorbidity, requiring a combined effort of both clinical (downstream) and policy (upstream) interventions, addressing the broader societal structures that contribute to health disparities [7, 28]. While most experts recognise the importance of including these broader system‐level factors in interventions and related research, they concurrently describe difficulties in their application and measurement because they cannot always be readily manipulated [32] and may fall outside of traditional clinical/health boundaries. This may explain the tendency to focus on individual‐level factors in interventions and research [32].

### 4.1 Limitations

This study provides insight into the experiences of individuals with comorbid physical health needs and SMI, but the findings should be considered in the context of some limitations. First, recruiting participants for this study proved to be challenging and resulted in a relatively small sample size. While we do not believe that this impacted the themes generated, as saturation in the data was achieved, it is possible that participants were different from individuals who did not participate. Specifically, our participants were likely in better health, given they actively followed through on participation and were recruited from secondary mental health services, meaning they were already engaged in receiving healthcare. Individuals who did not participate may have been too unwell to partake, not engaged in healthcare, or lacked trust in healthcare research due to past negative experiences, a common issue for individuals with SMI [33]. Our service user steering group suggested that a recruitment strategy based on community engagement, as well as the addition of members of the steering group as co‐researchers, may have expedited recruitment, enabled access to a richer sample and further refined our themes. However, project resources did not allow for this change in research design, which would have required a substantive amendment to the protocol and additional training. These considerations would need to be taken into account and planned for in the early stages of future research.

Furthermore, the Covid‐19 pandemic was a notable disruption for the study; focus groups could not proceed, interviews were moved to online formats, and recruitment became increasingly difficult. Despite starting the study before the Covid‐19 pandemic and continuing during the pandemic, specific additional challenges relating to physical healthcare during the pandemic did not arise in our interviews. Our study instead highlighted that the people we interviewed, with severe mental health conditions and physical health problems, experienced marked inequalities before the pandemic, which continued to be a concern during the pandemic. Our study was not designed to specifically examine the impact of the pandemic on people with severe mental health conditions; however, the themes raised are consistent with other qualitative and quantitative studies in this area [33, 34].

Finally, the research findings concerning service provision and structures are especially pertinent to the context of South East London, which is a relatively socio‐economically deprived and highly ethnically diverse urban borough. As a result, the findings regarding healthcare issues may not be applicable to other contexts. Further research across a range of settings and wider recruitment strategies based on partnerships with community organisations as well as mental health services is warranted to better understand how we can support individuals with comorbid physical health needs, SMI and other minoritised identities.

### 4.2 Implications and Conclusions

This study highlights systemic barriers and interpersonal stigma as sources of inequality for people with comorbid physical health needs and SMI. We make important suggestions for creating equitable conditions in healthcare for these individuals, including consistent care over solely crisis responses, clear communication and coordination between health professionals and services, and the important role of informal and formal social supports in maintaining overall health. The findings highlight the need for investment in social and healthcare roles, services and systems, which can be responsive to compounding risks experienced by individuals with comorbid physical health needs and SMI. Both physical and mental health needs, and their interactions, should be considered, along with the cumulative impact of poor socio‐economic conditions and stressors.

## Author Contributions

The study was conceptualised by J.D.M., S.P., and J.W. who also oversaw study design, data collection and analysis. T.K.O. led data analysis and drafting of the original draft of the manuscript for publication. All authors contributed to analysis and interpretation of the data and to reviewing and editing of the manuscript. All authors have approved the final version of the manuscript for publication and agree to be accountable for all aspects of the work. J.D.M. was responsible for funding acquisition.

## Ethics Statement

Ethics approval was obtained from the London‐Camberwell St Giles Research Ethics Committee (REC reference [18]/LO/0056) to conduct this study.

## Conflicts of Interest

The authors declare no conflicts of interest.

## Data Availability

The data that support the findings of this study are not publicly available due to privacy or ethical restrictions.

## Endnotes

1 EUPD = Emotionally unstable personality disorder.

2 PIP = Personal Independence Payment is a benefit in the United Kingdom for people who have a long‐term illness or disability.
